# The Rational Treatment of Consumption

**Published:** 1899-06-10

**Authors:** 


### THE RATIONAL TREATMENT OF CONSUMPTION.

In addressing the recent International Congress on Tuberculosis, Dr. Sinclair Coghill pointed out that it is impossible to treat pulmonary tuberculosis on scientific lines without having constant regard on the one hand to its pathogeny and the sequence of the different stages of its evolution, and, on the other, to the relations which exist between the symptoms and the pathological processes in which they have their origin. Pulmonary tuberculosis presents itself in two distinct stages—(1) the acute or pyrexial, and (2) the chronic. The former, which precedes the latter, has much the graver significance. It usually passes into the second, or it may return again and again in the course of the latter, indicating a repeated recurrence of destructive processes. Pyrexial phthisis must always be regarded as a specifically infective fever, which is from the beginning adynamic in type. In the earlier stage the pyrexia corresponds to the varying conditions within the lung of irritation, inflammation, and consolidation, and it is in this early acute stage that the various forms of local counter irritation are indicated, such as repeated small blisters when there is intercostal tenderness, or the actual cautery when the expectoration is blood-stained or pneumonic in character, or preparations of iodine applied externally when large areas of pleural tenderness call for relief.

The second stage of pyrexial phthisis has all the characters of a septic fever, and is characterised by an extreme diurnal range of temperature. It is, in fact, produced by the absorption of the necrotic material resulting from the progressing bacillary infection. The rational treatment of pyrexial phthisis may be considered under the three heads of general treatment, diet, and medicinal therapeutics. Absolute repose both of body and mind is important, and in extreme cases this may mean the maintenance of the horizontal position for a considerable length of time, although not necessarily in bed. The air breathed must be pure, and if possible the windows should be open day and night, warmth being maintained by the addition of light additional coverings rather than by raising the temperature of the apartment. The mind should be kept occupied by reading rather than by conversation. The whole body should be sponged over night and morning by the nurse with eau de Cologne, or spirits of wine and hot water, or with toilet vinegar. In regard to diet, a large meal of any kind or red meat at any time should not be permitted. Food should be given in small quantities at regular intervals—say, every two hours during the day, and three hours during the night. The food should be varied, and, as far as possible, the heavier meals should be made to alternate with lighter ones. Stimulants should be used with extreme caution, and may take the form of a tablespoonful of old Cognac beaten up with a fresh egg at intervals, or of a glass of dry champagne with a meat meal twice a day if the mouth is dry or the appetite is poor. Malt liquors and all red wines are more or less incompatible with a diet into which milk largely enters.

In regard to medicines, in view of the great importance of maintaining the efficiency of the digestive organs, it is often necessary to give drugs directed to the rectification of any errors of digestion which may arise. Then certain symptoms are sometimes of such urgency as to deserve special treatment. Cough, for example, may be relieved by the use of a mixture of chloroform and guaiacol on a respirator-inhaler. In regard to special or specific remedies Dr. Coghill refers especially to guaiacol and tuberculin. Guaiacol he administers hypodermically, in doses of 15 minims or more, combined with five minims of solution of strychnine, in cases where it is desirable to reduce the temperature. He also uses it in chronic cases in which the destruction of lung is extensive and the amount of expectoration is large. Tuberculin, however, is the only remedy which as yet has established any distinct claim to be considered as specific, and in regard to it Dr. Coghill does not hesitate to state his belief that the reaction of professional opinion against it has been as unwarrantable as its too sanguine and hasty announcement was unwise. He attributes his success with it to his using doses sufficiently small to avoid excessive reaction, which he looks upon as essentially destructive in character.

It will thus be seen that while Dr. Coghill recognises the advantages and the necessity of pure air in the treatment of consumption, he is very strongly of opinion that much more can be done for this disease than is included in mere dietetic and hygienic regimen. Speaking of the "expectant treatment as carried out more or less completely at some of the sanatoria for tuberculous patients, where it is professed that hygienic measures are alone employed," he says that "such an exclusive method is to be deprecated in the strongest terms," and is "a distinctly retrograde step in therapeutics." In other words, the appreciation of hygienic methods, which are good in themselves, has been carried too far, and there is now a tendency to neglect other not less valuable resources which are at our disposal. "To dispense with these therapeutic weapons is like a man combating a relentless enemy with his fists only while potent arms of precision lie neglected at his side." When, however, the chronic stage of the disease has been reached, and when the thermometric record has settled permanently at the normal, then the treatment becomes chiefly disciplinary and hygienic.

